# Sympathetic tone dictates the impact of lipolysis on FABP4 secretion

**DOI:** 10.1016/j.jlr.2023.100386

**Published:** 2023-05-10

**Authors:** Kacey J. Prentice, Alexandra Lee, Paulina Cedillo, Karen E. Inouye, Meric Erikci Ertunc, Jillian K. Riveros, Grace Yankun Lee, Gökhan S. Hotamisligil

**Affiliations:** 1 Department of Molecular Metabolism; Sabri Ülker Center for Metabolic Research, Harvard T.H. Chan School of Public Health, Boston, MA, USA; 2 Broad Institute of Harvard and MIT, Cambridge, MA, USA

**Keywords:** lipolysis, adipocytes, adipose tissue, adipose triglyceride lipase, fatty acid binding proteins, fatty acid transport, sympathetic tone

## Abstract

Levels of circulating fatty acid binding protein 4 (FABP4) protein are strongly associated with obesity and metabolic disease in both mice and humans, and secretion is stimulated by β-adrenergic stimulation both in vivo and in vitro. Previously, lipolysis-induced FABP4 secretion was found to be significantly reduced upon pharmacological inhibition of adipose triglyceride lipase (ATGL) and was absent from adipose tissue explants from mice specifically lacking ATGL in their adipocytes (ATGL^AdpKO^). Here, we find that upon activation of β-adrenergic receptors in vivo, ATGL^AdpKO^ mice unexpectedly exhibited significantly higher levels of circulating FABP4 as compared with ATGL^fl/fl^ controls, despite no corresponding induction of lipolysis. We generated an additional model with adipocyte-specific deletion of both FABP4 and ATGL (ATGL/FABP4^AdpKO^) to evaluate the cellular source of this circulating FABP4. In these animals, there was no evidence of lipolysis-induced FABP4 secretion, indicating that the source of elevated FABP4 levels in ATGL^AdpKO^ mice was indeed from the adipocytes. ATGL^AdpKO^ mice exhibited significantly elevated corticosterone levels, which positively correlated with plasma FABP4 levels. Pharmacological inhibition of sympathetic signaling during lipolysis using hexamethonium or housing mice at thermoneutrality to chronically reduce sympathetic tone significantly reduced FABP4 secretion in ATGL^AdpKO^ mice compared with controls. Therefore, activity of a key enzymatic step of lipolysis mediated by ATGL, per se, is not required for in vivo stimulation of FABP4 secretion from adipocytes, which can be induced through sympathetic signaling.

Fatty acid binding protein 4 (FABP4) is a recently identified adipokine and the only known adipokine to be secreted in response to lipolytic stimuli. Circulating levels of FABP4 are increased during fasting and suppressed by insulin (1, 2). Hormonal FABP4 has a strong positive correlation with BMI and is associated with numerous metabolic disorders, including diabetes, cardiovascular disease, and various types of cancers (3). Studies investigating the mechanism of action of hormonal FABP4 are yet to identify a defined receptor; however, they clearly demonstrate roles in numerous tissue types, including the liver to regulate hepatic glucose production, and the beta cell to influence insulin secretion (1, 4, 5). Thus, the coupling of FABP4 secretion with nutrient status likely indicates a role for FABP4 in the regulation of interorgan communication to coordinate energy and substrate fluxes and maintain metabolic health. However, in the context of obesity where lipolysis is unrestrained because of insulin resistance, levels of FABP4 are chronically elevated in the circulation (6, 7, 8). This aberrant FABP4 secretion stimulates glucose production from the liver, impairs beta-cell function, and potentiates the metabolic abnormalities associated with obesity (9). This role for hormonal FABP4 has been supported by studies using anti-FABP4 antibodies, which have demonstrated great therapeutic benefit in the treatment of both type 1 and type 2 diabetes in preclinical models (5, 10, 11).

Numerous studies have investigated the mechanisms of FABP4 secretion from adipocytes. We and others have demonstrated that FABP4 secretion is coupled to lipolytic stimuli, downstream of cAMP generation, and Ca^2+^ influx (2, 12, 13, 14). Consistent with this, genetic or pharmacological inhibition of adipocyte triglyceride lipase (ATGL), the first lipase involved in the lipolysis response that is responsible for the conversion of triglycerides (TGs) into diglycerides (15), abolishes FABP4 secretion from adipocyte cell lines (2). Inhibition of hormone-sensitive lipase (HSL), the second enzyme in the pathway, blunts FABP4 secretion to a lesser degree, suggesting that initiation of lipolysis is essential for FABP4 secretion (2). These findings have led to the hypothesis that FABP4 secretion is initiated by binding to the fatty acids liberated during the lipolytic process. However, the impact of lipid exposure to increase FABP4 secretion is rather limited compared with the effect of lipolytic signals (2), suggesting the potential existence of additional regulatory mechanisms. Once lipolysis signals are delivered, FABP4 is released from adipocytes in a nonclassical manner primarily through secretory lysosomes, as evidenced by inhibition of secretion by the lysosomal secretory inhibitor, chloroquine (16). A small fraction of FABP4 may also be released from adipocytes via a vesicular compartment (2).

Studies on the mechanisms of FABP4 secretion in vivo are very limited. In this study, we aimed to investigate the role of lipolysis in FABP4 secretion in vivo in a model of ATGL deficiency in adipocytes. Since ATGL is the most proximal catalytic step of the lipolytic process, its deficiency in adipocytes provides a system to investigate the requirement for the catalytic process in lipolysis-induced FABP4 secretion from these cells. Our results indicate that ATGL-mediated lipid breakdown in adipocytes is not necessary for FABP4 release into circulation in vivo, which can be triggered through sympathetic signaling.

## Materials and methods

### Animal care

Animal care and experimental procedures were performed with approval from the Harvard Medical School Standing Committee on Animals. Mice were studied at 8–10 weeks of age and were maintained on a 12 h light/12 h dark cycle in the Harvard T.H. Chan School of Public Health pathogen-free barrier facility with free access to water and to a standard laboratory chow diet (PicoLab Mouse Diet 20; catalog no.: 5058; LabDiet). ATGL^fl/fl^ mice on a C57BL/6 background (kind gift from Dr Erin Kershaw, University of Pittsburgh, Pittsburgh, PA) were crossed with Adiponectin-Cre mice to generate adipocyte-specific ATGL KO mice (ATGL^AdpKO^) and littermate floxed controls (17, 18). HSL^AdpKO^ and monoacylglycerol lipase (MGL)^AdpKO^ were similarly generated using HSL^fl/fl^ mice (kind gift from Dr Rudolf Zechner and Dr Günter Haemmerle, University of Graz, Austria) or MGL^fl/fl^ mice (kind gift from Dr Rudolf Zechner and Dr Robert Zimmerman, University of Graz, Austria). Generation of adipocyte-specific FABP4- ATGL-KO mice (double KO [DKO]) was accomplished by first crossing humanized FABP4 flox mice on C57BL/6 background (generated for the Hotamisligil laboratory by genOway) with ATGL^fl/fl^ animals. Homozygous FABP4^fl/fl^ATGL^fl/fl^ mice were then crossed with Adiponectin-Cre mice to generate DKO and FABP4^fl/fl^ATGL^fl/fl^ littermate controls. Genotyping for FABP4 flox, ATGL flox, and Cre recombinase was commercially outsourced and performed from tail biopsies or ear punches using real-time PCR (Transnetyx, Cordova, TN). Genotyping for HSL flox and MGL flox was performed as previously described (2). For evaluation of body composition, mice were anesthetized with isoflurane, and fat and lean mass measurements were performed using a DEXA scanner (PIXImus GE). For thermoneutral experiments, 8-week-old mice born at room temperature were housed in a Memmert Climate Chamber (Memmert HPP 750 LIFE Constant Climate Chamber) maintained at 30°C with 50% humidity on the same 12 h light-dark cycle for 2 weeks prior to in vivo lipolysis experiments and maintained for an additional week prior to tissue harvest.

### Adipose explant lipolysis

Perigonadal adipose tissue was collected from 6 h fasted 8- to 10-week-old male mice and washed with ice-cold PBS. Explants were minced with scissors to obtain pieces of 2–4 mm^3^. The pieces were washed five times with PBS, and five pieces were manually picked into each well of a 12-well plate. One milliliter of fresh serum-free DMEM was added to each well and incubated for 1 h at 37°C. Conditioned media were harvested for the assessment of basal/unstimulated secretion. Pieces were rinsed once with fresh DMEM, and DMEM containing forskolin (FSK; 1–3 μM, Cayman Chemical; catalog no.: 11018), isoproterenol (ISO; 10 μM, Tocris Biochemical; catalog no.: 1747), or norepinephrine (NE; 1 μM, Sigma; catalog no.: A9512) were added to stimulate lipolysis. The plates were incubated for 1 h at 37°C, after which conditioned media were collected for measurement of FABP4 and glycerol. Adipose tissue pieces in each well were then lysed for normalization of secretion to total protein. Conditioned media were centrifuged at 5,000 rpm for 10 min, and the supernatant was used for subsequent assays.

### In vivo lipolysis

Lipolysis was induced in 8- to 10-week-old mice using the pan-beta adrenergic agonist, ISO or the beta 3-specific agonist, CL-316,243. Mice were fasted beginning at 9 a.m. for 6 h, and lipolysis was induced by injection of freshly prepared and sterile-filtered ISO (10 mg/kg, IP, Tocris Bioscience, catalog no.: 1747) or CL-316,243 (0.1 mg/kg, IP, Tocris Bioscience, catalog no.: 1499) in PBS. About 70 μl of blood was collected from a tail nick into heparinized capillary tubes at baseline (0) before injection, and 15, 30, and 60 min after injection, for measurement of plasma FABP4, insulin, NEFA, corticosterone, and glycerol levels. For studies using hexamethonium (Hex), mice were injected IP with 20 mg/kg Hex (Tocris, catalog no.: 4111) or PBS 30 min prior to induction of lipolysis with CL-316,243. For thermoneutral experiments, mice were housed in the temperature-controlled chamber at 30°C throughout the lipolysis experiment, and blood was collected from mice under a heat lamp to prevent activation of thermogenic pathways. For FABP4 clearance studies, 10 μg of recombinant mouse FABP4 (produced in-house) was injected intraperitoneally into mice, and blood collected via tail vein at 10, 20, 60, and 120 min postinjection.

### Cell culture

3T3-L1 preadipocytes (Zen-Bio SP-L1-F) were maintained in DMEM with 10% bovine calf serum. For differentiation, cells were seeded in 12-well plates and grown to confluency. Adipocyte differentiation was induced in DMEM with 10% FBS using 500 μM 3-isobutyl-1-methylxanthine (Sigma; catalog no.: I5879), 5 μg/ml insulin (Sigma; catalog no.: I9278), 1 μM dexamethasone (Sigma; catalog no.: D4902), and 2 μM rosiglitazone (Cayman; catalog no.: 71740) for 48 h, after which the cells were fed fresh DMEM with 10% FBS and 5 μg/ml insulin every 2 days. Lipolysis assays were performed on days 10–12 of induction. For experiments involving atglistatin (Sigma; catalog no.: SML1075), cells were pretreated with 10 μM in DMEM for 2 h. For induction of lipolysis, adipocytes were washed three times in PBS and incubated in serum-free DMEM for 1 h at 37°C to evaluate basal secretion. Cells were rinsed with serum-free DMEM, and lipolysis was induced by addition of DMEM with 1 μM FSK, 10 μM ISO, or 10% serum from treated mice for 1 h at 37°C. Conditioned media were collected at the end of each time point, centrifuged at 5,000 rpm for 10 min, and supernatant was used for further assays. For mouse serum assays, mice were injected intraperitoneally with either PBS or 10 mg/kg ISO. After 10 min, mice were euthanized by CO_2_, and total blood was collected via cardiac puncture. FABP4 was evaluated in the serum as harvested from the mice. Serum was then diluted in DMEM to a final concentration of 10% and added to cells for 1 h. FABP4 was quantified in the 10% serum media before addition to the cells and in the conditioned media following incubation. The final graph is from conditioned media, corrected for the FABP4 in the media containing 10% serum (value subtracted).

### Protein extraction, SDS-PAGE, and Western blotting

Mice were fasted for 6 h before euthanasia and tissue harvesting. Tissues were snap frozen in liquid nitrogen until protein extraction was performed. Whole adipose tissue was homogenized in ice-cold RIPA buffer (Cell Signaling Technologies; catalog no.: 9806) containing 2 mM activated Na_3_VO_4_ and 1% protease inhibitor cocktail (Sigma; catalog no.: P340). The homogenate was centrifuged at 15,000 rpm at 4°C for 25 min to pellet the cellular debris. The resulting supernatant was assayed for protein concentration by BCA assay (Thermo Fisher/Pierce; catalog no.: 23225). Lysates were diluted with 4× Laemmli buffer containing beta-mercaptoethanol, boiled for 10 min at 95°C, and subjected to SDS-polyacrylamide gel electrophoresis 4–20% (Criterion TGX Stain-Free Protein Gel; Bio-Rad) gels. Protein was transferred to PVDF membrane using Bio-Rad Transblot Turbo semidry system. Membranes were blocked for at least 1 h in TBS with Tween with 5% protease-free BSA. Detection antibodies were diluted in TBS with Tween with 5% protease-free BSA and incubated on membranes overnight at 4°C. Proteins of interest were detected using 1:1,000 dilutions of ATGL antibody (CST; catalog no.: 2138), HRP-tagged anti-FABP4 antibody (clone 351.4.5E1.H3, generated in house for Hotamisligil laboratory by Dana Farber Antibody Core), or HRP-tagged anti-β-actin antibody (CST; catalog no.: 5125) as a loading control. Ponceau S staining was used as a loading control for thermoneutral experiments. PVDF membranes were washed three times with water for 10 min each and then incubated for 2 min in 20% methanol. Membranes were then placed in Ponceau S solution (Sigma; catalog no.: 7170) for 5 min and rinsed with water before imaging. FABP4 antibody has been validated with protein lysates from FABP4-KO mice as negative control and recombinant FABP4 as positive control. HRP signal was detected with chemiluminescent substrate (Pierce SuperSignal West Pico or Femto Plus, Thermo Fisher; catalog no.: 34580 or 34094).

### Plasma and media assays

Plasma and media FABP4 were measured by in-house FABP4 ELISA using anti-FABP4 antibodies produced for the Hotamisligil laboratory by the Dana Farber Antibody Core (clone 351.4.2E12.H1.F12 for capture and HRP-tagged clone 351.4.5E1.H3 for detection) and recombinant human FABP4 (R&D Systems; catalog no.: DY3150-05) or recombinant mouse FABP4 (Biovendor; catalog no.: RD191036200R) as a standard. Plasma insulin was assessed using a Crystal Chem Ultra-Sensitive Mouse Insulin ELISA (catalog no.: 90080). Plasma and media NEFA and glycerol were measured by enzymatic colorimetric assays (FujiFilm, Wako HR Series NEFA-HR(2); catalog nos.: 999-34691, 995-34791, 991-34891, 993-35191; Sigma Free Glycerol Reagent, catalog no.: F2468). Corticosterone was evaluated using Abcam Corticosterone ELISA (catalog no.: ab108821).

### Insulin and glucose tolerance tests

Before intraperitoneal glucose tolerance test or oral glucose tolerance test (OGTT), mice were fasted 16 h overnight, before injection or gavage of 0.75 g/kg body weight of glucose. Insulin tolerance tests (ITTs) were performed following 4 h fasting (10 a.m.–2 p.m.). Mice were injected intraperitoneally with 0.25 IU/kg body weight of recombinant human insulin (Humulin-R; Eli Lilly) prepared in sterile PBS with 0.1% BSA. For acute injection tests, mice were fasted for 4 h followed by intraperitoneal injection of 150 μl of sterile PBS. For all tests, blood glucose was evaluated at 0, 15, 30, 45, 60, 90, and 120 min postinjection or gavage. Blood samples were collected from a tail nick into heparinized capillary tubes for evaluation of FABP4 and insulin levels.

### Statistics

Statistical analysis was performed using GraphPad Prism, version 9.4.0 for MacOS (GraphPad Software, San Diego, CA; www.graphpad.com). All data are presented as mean ± SEM.

## Results

### Impact of ATGL on FABP4 secretion in vitro and in vivo

**Fig. 1:**
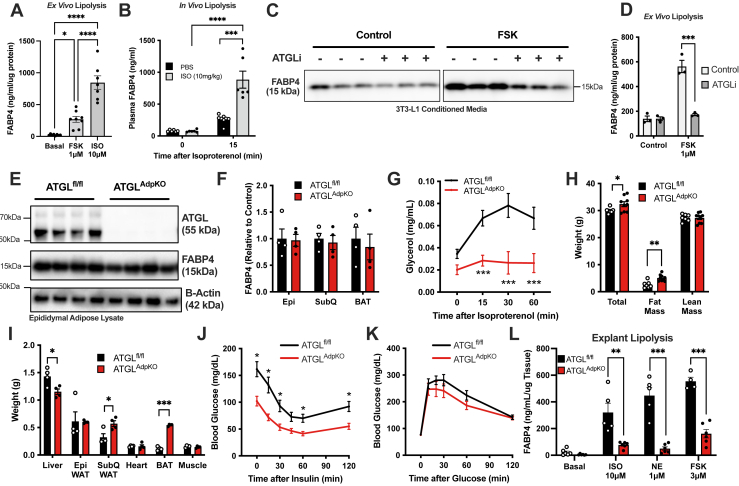
FABP4 secretion is dependent on lipolysis in vitro. A: FABP4 secretion from wild-type epididymal adipose tissue explants under unstimulated (basal) conditions or induction of lipolysis by FSK (1 μM) or ISO (10 μM) (N = 7–8/condition; one-way ANOVA). B: Plasma FABP4 in wild-type mice following injection of PBS or ISO (10 mg/kg) (N = 6/group; two-way ANOVA). FABP4 in conditioned media from (C) 3T3-L1 adipocytes (Western blot; FSK 10 μM) and (D) epididymal adipose tissue explants (ELISA quantification) following 2 h pretreatment with atglistatin (ATGLi; 10 μM) (N = 3/group; two-way ANOVA). E: Western blot of epididymal adipose tissue confirming ATGL deletion in ATGL^AdpKO^ (N = 4/group). F: Quantification of FABP4 in adipose depots from ATGL^fl/fl^ and ATGL^AdpKO^ mice (N = 4/group; Student’s *t*-test). G: Plasma glycerol following induction of lipolysis in vivo (N = 14–16/group; two-way ANOVA). H: Body compo as determined by dual-energy X-ray absorptiometry (DEXA) (N = 7–9/group; Student’s *t*-test). I: Weight of liver, epididymal (Epi) adipose tissue, subcutaneous (SubQ) adipose tissue, heart, brown adipose tissue (BAT), and gastrocnemius muscle (N = 4/group; Student’s *t*-test). Glucose excursion curves in (J) TT and (K) intraperitoneal glucose tolerance test (ipGTT) (N = 9–10/group; two-way ANOVA). L: FABP4 secretion from epididymal adipose tissue explants under unstimulated (basal) conditions or induction of lipolysis by ISO (10 μM), NE (1 μM) or FSK (3 μM) (N = 4–6/condition; Student’s *t*-test). All mouse experiments performed in 8- to 10-week-old male mice. Data are presented as mean ± SEM.

It is well established that lipolytic stimuli robustly increase FABP4 secretion from adipocytes in vitro, ex vivo, and in vivo (2, 12, 13, 14, 16). Activation of beta-adrenergic receptors using ISO, or direct stimulation of cAMP generation through activation of adenylyl cyclase using FSK, potently stimulated lipolysis and FABP4 release from epididymal adipose tissue explants (Fig. 1A). Similarly, injection of ISO into wild-type mice robustly elevated plasma FABP4 levels (Fig. 1B). Consistent with previous reports, induction of FABP4 secretion from adipocytes was dependent on ATGL activity, as treatment of 3T3-L1 adipocytes (Fig. 1C) or adipose explants (Fig. 1D) with atglistatin, an inhibitor of ATGL, the enzyme that catalyzes the first step of TG breakdown, consistently prevented lipolysis-induced FABP4 secretion in vitro and ex vivo (2).

To gain insight into the relationship between FABP4 secretion and lipolysis in vivo, we generated a mouse model of adipocyte-specific ATGL deficiency (ATGL^AdpKO^) by crossing Adiponectin-Cre-expressing mice to ATGL^fl/fl^ animals. We validated the absence of ATGL protein in epididymal, subcutaneous, and brown adipose depots by Western blot analysis (Fig. 1E; epididymal adipose representative). Importantly, ATGL deficiency did not impact FABP4 protein levels in any of the depots examined (Fig. 1F). The absence of ATGL activity was confirmed by a lack of induction of lipolysis, as evaluated by plasma glycerol, following ISO injection (Fig. 1G). Consistent with previous reports (17, 19), ATGL^AdpKO^ mice exhibited only a minor increase in body weight, attributed to increased fat mass, with no change in total lean mass (Fig. 1H). Specifically, we observed increases in subcutaneous and brown fat depots, with no change in epididymal fat mass, and a reduction in liver weight (Fig. 1I). Despite a minor increase in adiposity, ATGL^AdpKO^ mice exhibited a significant improvement in insulin sensitivity, as assessed by ITT (Fig. 1J). Furthermore, upon intraperitoneal glucose tolerance test, ATGL^AdpKO^ mice had a minor improvement in overall glucose tolerance (Fig. 1K). Consistent with our previous observations (2), epididymal adipose tissue explants from ATGL^AdpKO^ mice failed to secrete FABP4 in response to lipolytic stimuli ISO, FSK, or NE ex vivo (Fig. 1L).

### Adipocyte FABP4 secretion is increased in ATGL^AdpKO^ mice in vivo

**Fig. 2:**
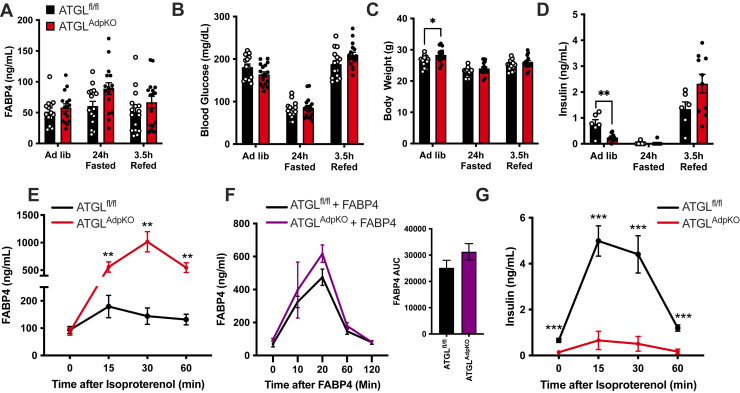
Adipocyte-specific ATGL KO mice have enhanced FABP4 secretion in vivo. Quantification of (A) plasma FABP4, (B) blood glucose, (C) body weight, and (D) plasma insulin in ATGL^fl/fl^ (black bars) and ATGL^AdpKO^ (red bars) mice under ad libitum-fed conditions, following 24 h food withdrawal, and 3.5 h after food was added back to cages (N = 15–17/group; two-way ANOVA). E: Plasma FABP4 in ATGL^fl/fl^ and ATGL^AdpKO^ following induction of lipolysis with ISO (10 mg/kg) (N = 15–16/group; two-way ANOVA). F: Plasma FABP4 levels in ATGL^fl/fl^ and ATGL^AdpKO^ mice injected with 10 μg recombinant FABP4 (N = 3–5/group; two-way ANOVA). G: Plasma insulin in ATGL^fl/fl^ and ATGL^AdpKO^ following induction of lipolysis with ISO (10 mg/kg) (N = 15–16/group; two-way ANOVA). All mouse experiments performed in 8- to 10-week-old male mice. Data are presented as mean ± SEM.

To evaluate if FABP4 secretion is indeed altered in the ATGL^AdpKO^ mice, consistent with in vitro and ex vivo observations, we determined circulating FABP4 levels in response to fasting. Unexpectedly, 24 h fasted ATGL^AdpKO^ mice did not exhibit a defective induction of FABP4 secretion as compared with ATGL^fl/fl^ controls (Fig. 2A). This was in the context of similar blood glucose levels and body weight following fasting (Fig. 2B, C). In line with improved insulin sensitivity, ATGL^AdpKO^ mice exhibited reduced plasma insulin levels when fed ad libitum; however, there were no differences in plasma insulin in either the fasted or refed states (Fig. 2D). Next, we examined FABP4 secretion in these mice upon administration of ISO to stimulate lipolysis. Strikingly, ATGL^AdpKO^ mice exhibited a marked increase in plasma FABP4 concentrations in response to lipolysis stimulation as compared with the ATGL^fl/fl^ controls, despite no induction of lipolytic products (Figs. 2E and 1G). To evaluate if increased plasma FABP4 levels are associated with increased secretion or reduced clearance, we injected ATGL^fl/fl^ and ATGL^AdpKO^ mice with 10 μg of recombinantly produced FABP4 and monitored plasma clearance. There were no differences in FABP4 levels between groups at any timepoints examined, with both groups returning to baseline levels 2 h postinjection, suggesting increased plasma FABP4 levels in ATGL^AdpKO^ mice are due to increased secretion and not impaired clearance (Fig. 2F). Insulin can act to suppress lipolysis and has previously been shown to suppress FABP4 secretion in vitro (2). Interestingly, upon stimulation of lipolysis, ATGL^AdpKO^ mice had a significantly blunted insulin secretion response (Fig. 2G), which may be due to their improved insulin sensitivity. These findings raise multiple possibilities for the mediation of hormonal FABP4 secretion in response to ISO in ATGL^AdpKO^ mice in vivo, including the possibility of a nonadipocyte cell source, a lack of suppression by insulin, or the lack of lipolytic products potentiating compensatory FABP4 release.

**Fig. 3:**
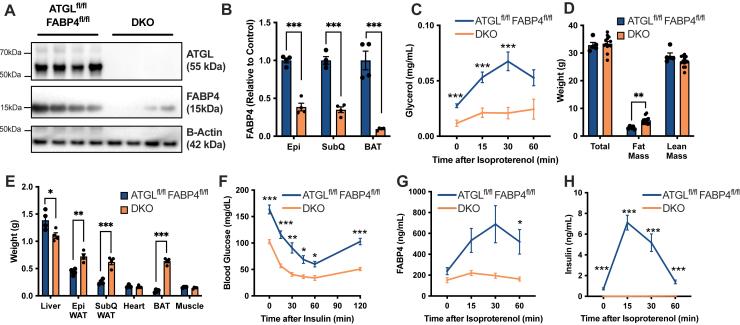
Lipolysis-induced FABP4 secretion is from adipocytes in vivo. A: Western blot of epididymal adipose tissue confirming ATGL and FABP4 deletion in FABP4/ATGL^AdpKO^ (DKO) mice (N = 4/group). B: Quantification of FABP4 in adipose depots from ATGL^fl/fl^ FABP4^fl/fl^ and DKO mice (N = 4/group; Student’s *t*-test). C: Plasma glycerol following induction of lipolysis in vivo (N = 13–15/group; two-way ANOVA). D: Body composition as determined by dual-energy X-ray absorptiometry (DEXA) (N = 5–13/group; Student’s *t*-test). E: Weights of liver, epididymal (Epi) adipose tissue, subcutaneous (SubQ) adipose tissue, heart, brown adipose tissue (BAT), and gastrocnemius muscle (N = 4/group; Student’s *t*-test). F: Glucose excursion curves in ITT (N = 6–9; two-way ANOVA). G: Plasma FABP4 and (H) plasma insulin in ATGL^fl/fl^ FABP4^fl/fl^ and DKO mice following induction of lipolysis with ISO (10 mg/kg) (N = 14–15/group; two-way ANOVA). All mouse experiments performed in 8- to 10-week-old male mice. Data are presented as mean ± SEM.

FABP4 was originally characterized as an adipocyte-specific FABP; however, it is now well established that it is expressed in other cell types, including endothelial cells, bronchial epithelial cells, and macrophages (20, 21, 22, 23, 24). Given the stark contrast between in vivo and ex vivo FABP4 secretion in the ATGL^AdpKO^ mice, we investigated whether the in vivo lipolysis-induced FABP4 secretion in ATGL^AdpKO^ mice may be coming from a nonadipocyte cell source. Therefore, we crossed FABP4^fl/fl^ mice with the ATGL^fl/fl^ mice to generate FABP4^fl/fl^ATGL^fl/fl^ animals. These mice were then crossed with the Adiponectin-Cre line to generate FABP4/ATGL^AdpKO^ mice, hereafter referred to as DKO, lacking both FABP4 and ATGL specifically in adipocytes. Western blot analysis of epididymal, subcutaneous, and brown adipose depots again confirmed the deletion of ATGL and a significant reduction in FABP4 expression (Fig. 3A, B). The residual FABP4 expression is likely because of nonadipocyte cell types present in explants, including endothelial cells. As observed in the ATGL^AdpKO^ mice, the DKO animals exhibited a blunted lipolysis response, confirming deletion of ATGL (Fig. 3C). Similar to ATGL^AdpKO^ mice, DKO animals also exhibited a significant increase in fat mass, despite no differences in total body weight and lean mass (Fig. 3D). This increase in fat mass corresponded to significant increases in both epididymal and subcutaneous adipose depots, as well as increased brown adipose mass, with a reduction in liver weight (Fig. 3E). As observed in ATGL^AdpKO^, these mice exhibited a significant improvement in insulin sensitivity by ITT (Fig. 3F). When we stimulated lipolysis in the DKO animals by ISO injection, there was reduced induction of FABP4 in DKO mice compared with controls, confirming that the source of elevated FABP4 secretion in ATGL^AdpKO^ mice is indeed from adipocytes (Fig. 3G). Consistent with both ATGL^AdpKO^ mice, and our previous findings in whole-body FABP4-KO mice (4), there was blunted induction of insulin secretion in the DKO animals in response to lipolysis (Fig. 3H).

**Fig. 4:**
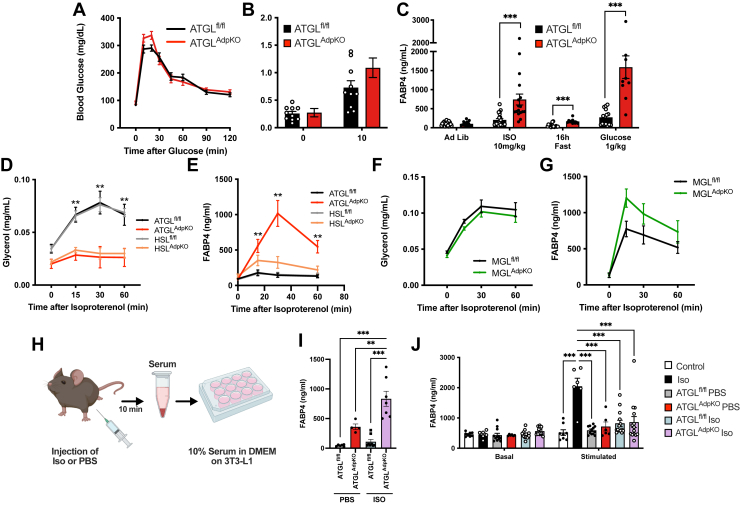
Glucose bolus stimulates FABP4 secretion in vivo. A: Glucose excursion curve during OGTT (N = 9–10/group; two-way ANOVA). B: Corresponding plasma insulin levels during OGTT (N = 9–10/group; two-way ANOVA). C: Plasma FABP4 in ATGL^fl/fl^ and ATGL^AdpKO^ mice under ad libitum fed, 10 min following ISO injection (10 mg/kg), 16 h fasting, and 10 min following glucose injection (1 g/kg) (N = 10–21/group; Student’s *t*-test). D: Plasma glycerol and (E) plasma FABP4 levels in ATGL^AdpKO^ and HSL^AdpKO^ and respective floxed control mice injected with ISO (10 mg/kg) (N = 9–16/group; two-way ANOVA). F: Plasma glycerol and (G) plasma FABP4 levels in MGL^fl/fl^ and MGL^AdpKO^ mice injected with ISO (10 mg/kg) (N = 5/group; two-way ANOVA). H: Schematic illustration of the protocol for serum induction of lipolysis in 3T3-L1 cells. I: Plasma FABP4 levels in mice 10 min following injection of PBS or ISO (10 mg/kg) (N = 4–7/group; one-way ANOVA). J: Quantification of FABP4 in conditioned media from 3T3-L1 adipocytes treated with control, ISO (1 μM), or 10% serum from mice in (I) (N = 6–14/group; one-way ANOVA). All mouse experiments performed in 8- to 10-week-old male mice. Data are presented as mean ± SEM.

Insulin acts to suppress lipolysis through a mechanism involving reduction of intracellular cAMP, the downstream messenger activated by ISO or FSK treatment (25, 26). Potentiation of adipocyte FABP4 secretion in response to lipolysis in vivo could be due to the lack of ISO-induced insulin secretion in the ATGL^AdpKO^ mice. Unfortunately, because of the extreme insulin sensitivity of the ATGL^AdpKO^ mice, our ability to supplement insulin during conditions of lipolysis to test this question is limited. While insulin secretion was blunted in response to ISO in the ATGL^AdpKO^ mice, we observed a normal potentiation of insulin secretion in response to glucose during an OGTT, with no difference in glucose tolerance (Fig. 4A, B). When we quantified plasma FABP4 10 min after glucose gavage, we observed a significant potentiation of FABP4 secretion in both ATGL^fl/fl^ and ATGL^AdpKO^ mice (Fig. 4C). Despite no difference in insulin secretion during OGTT, ATGL^AdpKO^ mice again secreted significantly more FABP4 than ATGL^fl/fl^ controls. Therefore, enhanced adipocyte FABP4 secretion in ATGL^AdpKO^ mice cannot be explained by a lack of insulin.

Previously, we demonstrated that inhibition of HSL or a combination of HSL and the final enzyme of the lipolysis cascade, MGL, suppressed FABP4 secretion in vitro, albeit to a lesser degree than that observed with ATGL inhibition (2). To determine if the absence of lipolytic products may be related to enhanced FABP4 secretion in ATGL^AdpKO^ mice in vivo, we evaluated lipolysis and FABP4 secretion in both HSL^AdpKO^ and MGL^AdpKO^ mice with respective HSL^fl/fl^ and MGL^fl/fl^ controls. Both ATGL^AdpKO^ and HSL^AdpKO^ mice exhibited nearly identical blunting of their plasma glycerol responses to ISO injection, indicating a lack of lipolysis and lipolytic products (Fig. 4D). However, while FABP4 secretion was potentiated in HSL^AdpKO^ mice, it was not nearly to the same degree as observed in ATGL^AdpKO^ mice (Fig. 4E), indicating that increased FABP4 secretion is not likely simply because of a lack of lipolytic products. MGL^AdpKO^ mice did not exhibit any differences in plasma glycerol or FABP4 levels as compared with MGL^fl/fl^ controls (Fig. 4F, G). To evaluate if there may be another circulating factor induced by ISO injection in ATGL^AdpKO^ mice in vivo that acts to potentiate adipocyte FABP4 release, we injected ATGL^AdpKO^ mice and ATGL^fl/fl^ controls with PBS or ISO and collected total serum via cardiac puncture 10 min later (illustrated in Fig. 4H). We again validated the presence of increased FABP4 levels in ATGL^AdpKO^ animals upon injection of either PBS or ISO (Fig. 4I). Media containing 10% serum from these mice were then added to differentiated 3T3-L1 adipocytes, and FABP4 secretion was evaluated, corrected for the concentration of FABP4 in serum-containing media. There was no difference in the levels of FABP4 secretion between 3T3-L1 cells treated with serum from ATGL^fl/fl^ or ATGL^AdpKO^ mice (Fig. 4J). These data suggest that there may not be a clear circulating factor responsible for increased FABP4 release in the absence of adipocyte ATGL.

### FABP4 secretion is potentiated by high sympathetic tone independent of lipolysis

**Fig. 5:**
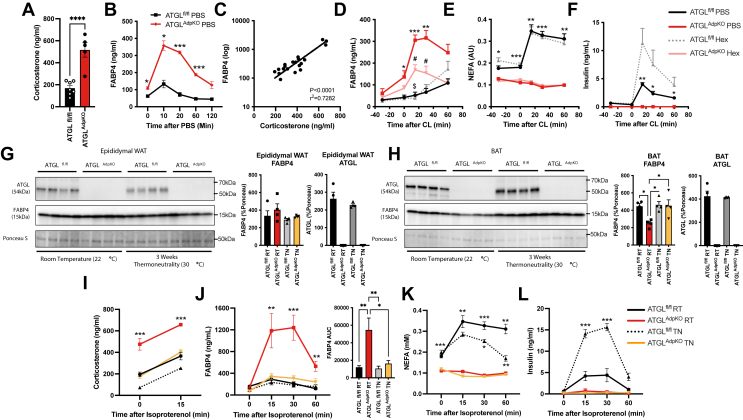
In vivo FABP4 secretion is blunted upon sympathetic nervous inhibition. A: Plasma corticosterone levels in 6 h fasted ATGL^fl/fl^ and ATGL^AdpKO^ mice (N = 5–7/group; Student’s *t*-test). B: Plasma FABP4 in ATGL^fl/fl^ and ATGL^AdpKO^ injected with 100 μl sterile PBS (N = 3–5/group; two-way ANOVA). C: Correlation of plasma FABP4 and plasma corticosterone levels in ATGL^fl/fl^ and ATGL^AdpKO^ mice (N = 20). D: Plasma FABP4, (E) NEFAs, and (F) insulin following administration of 20 mg/kg hexamethonium and 0.1 mg/kg CL-316,243 (N = 5–6/group; two-way ANOVA). Western blot and quantification of ATGL and FABP4 protein levels, normalized to total protein (Ponceau S stain), in (G) epididymal white adipose tissue (WAT) and (H) brown adipose tissue (BAT) from ATGL^fl/fl^ and ATGL^AdpKO^ housed at room temperature or 3 weeks at thermoneutrality (N = 4/group; one-way ANOVA). I: Plasma corticosterone levels in 6 h fasted ATGL^fl/fl^ and ATGL^AdpKO^ mice housed at room temperature or 2 week thermoneutrality and 15 min post ISO injection (10 mg/kg) (N = 6/group; two-way ANOVA). Plasma (J) FABP4, (K) NEFA, and (L) insulin following administration of ISO (N = 6/group; two-way ANOVA). All mouse experiments performed in 8- to 10-week-old male mice. Data are presented as mean ± SEM.

Finally, we hypothesized that the in vivo signal regulating FABP4 release from adipocytes upon ISO injection that is absent in ex vivo conditions may be related to sympathetic innervation of the tissue. ATGL^AdpKO^ mice have been previously reported to exhibit enhanced sympathetic tone, accompanied by significantly increased corticosterone levels (27), which can also potentiate sympathetic activity (28, 29). In agreement, we observed a 3-fold increase in plasma corticosterone levels in 6 h-fasted ATGL^AdpKO^ mice (Fig. 5A). Consistent with enhanced sympathetic activity, injection of PBS alone potently stimulated FABP4 secretion in ATGL^AdpKO^ animals, suggesting a powerful stress-induced response (Figs. 4I and 5B). When plasma FABP4 levels were plotted against plasma corticosterone levels, we observed a strong correlation (*P* < 0.0001, *r*^2^ = 0.7282), further supporting this hypothesis (Fig. 5C).

To evaluate if reducing sympathetic activity in ATGL^AdpKO^ mice would suppress FABP4 secretion, we utilized acute and chronic inhibition strategies. We previously demonstrated that FABP4 secretion upon exposure to propionic acid in vivo is diminished when sympathetic signaling is pharmacologically inhibited (30). Using this approach, we began by acutely inhibiting sympathetic signaling by pretreating mice with Hex, a nondepolarizing ganglionic blocker acting upstream of adrenergic receptors (31), 30 min prior to CL-316,243 injection. Remarkably, FABP4 secretion was reduced by more than 50% upon Hex administration in ATGL^AdpKO^ mice compared with vehicle-treated ATGL^AdpKO^ mice (Fig. 5D). Importantly, Hex treatment had no effect on the induction of lipolysis in ATGL^fl/fl^ mice (Fig. 5E). Insulin secretion was also not altered with Hex treatment in the ATGL^AdpKO^ mice but was significantly elevated in ATGL^fl/fl^ mice (Fig. 5F).

To evaluate the impact of chronic reduction in sympathetic activity in ATGL^AdpKO^ mice on FABP4 secretion, we housed mice at thermoneutrality (30°C) for 2 weeks beginning at 8 weeks of age. The elimination of cold stress associated with housing at room temperature conditions (22°C) is established to reduce sympathetic drive, evidenced by reduced tyrosine hydroxylase expression and reduced NE turnover (32, 33). There was no difference in FABP4 levels in white or brown adipose depots between ATGL^AdpKO^ and ATGL^fl/fl^ controls following 2 weeks of housing at thermoneutrality (Fig. 5G, H). However, there was a significant reduction in corticosterone levels, with ATGL^AdpKO^ thermoneutral levels matching room temperature ATGL^fl/fl^ controls (Fig. 5I). Strikingly, ISO-induced FABP4 secretion from ATGL^AdpKO^ thermoneutral mice decreased to become indistinguishable from ATGL^fl/fl^ controls (Fig. 5J). This corresponded to a paralleled corticosterone response to injection between ATGL^AdpKO^ thermoneutral mice and ATGL^fl/fl^ controls at room temperature (Fig. 5I). Intriguingly, NEFAs decreased significantly faster in ATGL^fl/fl^ mice under thermoneutrality compared with room temperature conditions (Fig. 5K). As expected, there was no induction in lipolysis in ATGL^AdpKO^ under either condition. Similar to observations with Hex treatment, insulin secretion was also increased in ATGL^fl/fl^ thermoneutral mice upon lipolysis stimulation but unchanged in ATGL^AdpKO^ mice, consistent with a role for sympathetic signaling in suppression of lipolysis-stimulated insulin secretion (Fig. 5L). Overall, the enhanced FABP4 secretion from ATGL^AdpKO^ mice upon β-adrenergic stimulation in vivo is likely because of elevated sympathetic tone (27) resulting in altered adipocyte signaling and potentiation of FABP4 release, an effect that is absent ex vivo.

## Discussion

FABP4 is secreted from adipocytes upon exposure to lipolytic stimuli in vitro. Under conditions of obesity, loss of insulin sensitivity in adipocytes results in unsuppressed lipolysis, which leads to increased levels of hormonal FABP4. Circulating FABP4 levels exhibit a strong positive correlation with diabetes and cardiovascular disease, and multiple independent genome-wide association studies have identified protection in individuals with a low-expression variant of FABP4 against type 2 diabetes and cardiovascular diseases (34, 35, 36). Importantly, targeting hormonal FABP4, either through genetic ablation or monoclonal antibody therapy, has been proven to protect against or reverse diabetes in preclinical models of genetic or diet-induced obesity (5, 9, 10). Therefore, understanding the mechanisms underlying the secretion and regulation of this hormone is of critical importance to realize its broad utility in multiple human diseases.

Previous work has provided an understanding of the in vitro and ex vivo mechanisms regulating FABP4 secretion from adipocytes. Increases in cAMP and intracellular calcium levels, downstream of β_3_-adrenergic receptor activation, are essential for the potentiation of FABP4 release (2, 12, 13). We have previously reported that the enzymes of lipolysis, ATGL and, to a lesser extent, HSL, are required for FABP4 secretion ex vivo and in vitro, as inhibition of ATGL either genetically or pharmacologically abolished secretion (2). Supportive of the role for the lipolytic process in the secretion of FABP4, inhibition of HSL or MGL, the lipolytic enzymes required for the second and third steps of TG breakdown, respectively, had decreasing efficacy at reducing FABP4 secretion, suggesting that initiation of lipolysis was essential for this process. Here, however, while we have shown that lipolysis is required for ex vivo secretion of FABP4, we speculate that there may be alternative pathways regulating secretion in vivo.

Our studies in the adipocyte-specific ATGL-deficient mouse have demonstrated that one such signal promoting FABP4 secretion comes from the sympathetic system, in vivo. Blocking sympathetic signaling using Hex or reducing sympathetic tone through thermoneutral housing resulted in significantly reduced FABP4 secretion in the context of adipocyte ATGL deficiency, indicating that this is a critical pathway regulating secretion in vivo. Importantly, human obesity is also characterized by an increase in sympathetic tone (37), which may also contribute to the observed correlation between hormonal FABP4 and BMI (23), and exacerbate the pathological elevations of circulating FABP4. Sympathetic tone is enhanced by glucocorticoids (28, 29), and ATGL^AdpKO^ mice exhibit significantly greater plasma corticosterone levels, correlating with significantly enhanced FABP4 secretion. Similarly, levels of FABP4 are significantly increased in individuals with Cushing’s disease compared with BMI-matched controls and demonstrate a positive correlation with urinary free cortisol (38), suggesting this mechanism of regulation of secretion is conserved in humans. The role for sympathetic activation in FABP4 release may be related to a role for this protein in mediating so-called “fight or flight” responses, transporting fatty acids, potentiating hepatic glucose production, and suppressing insulin secretion to facilitate survival (3). A recent study by Ron *et al.* (39) demonstrates that FABP4 levels are increased in neonates shortly after birth along with counterregulatory hormones such as cortisol and glucagon and is required for activation of gluconeogenesis and maintenance of glucose homeostasis following disconnection from the placental nutrient supply. It is possible that the lack of availability of the products of lipolysis upon β-adrenergic stimulation in the ATGL^AdpKO^ mice is a source of stress in this model, potentiating the release of FABP4. It is of importance to note that HSL^AdpKO^ mice do not exhibit cold intolerance as observed in ATGL^AdpKO^ mice and are therefore not anticipated to have the same degree of sympathetic activation (40, 41). This corresponds to reduced FABP4 secretion in HSL^AdpKO^ as compared with ATGL^AdpKO^ despite a similar absence in lipolytic products. Consistent with stress-induced secretion, potentiated FABP4 release upon glucose gavage in ATGL^AdpKO^ mice is likely associated with stress of prolonged 16 h fasting together with the stress of gavage. The potent stimulation of FABP4 secretion upon PBS injection alone in ATGL^AdpKO^ animals is further supported for this stress-induced secretion model.

It is also of interest that the ATGL-deficient animals remained insulin sensitive despite high levels of FABP4 in circulation. In this particular model, while the lipolytic signal is delivered to trigger FABP4 release, it occurs in the absence of lipolytic process and any associated lipid or other products. Given the strong link between hormonal FABP4 and a multitude of chronic and metabolic diseases in both mice and humans, it is possible that there are different functional roles for FABP4 depending on the cellular source and the lipid environment in the biological functions of this hormone. This is an intriguing system to further explore the mechanisms and functional relevance of apo- versus holo-FABP4 in vivo in future studies.

## Data availability

Source data for this article is publically available at doi:10.6084/m9.figshare.23197985.

## Conflict of interest

G. S. H. is in the Scientific Advisory Board of Crescenta Biosciences and holds equity. The Hotamisligil laboratory holds intellectual property related to hormonal FABP4 and its therapeutic targeting. All other authors declare that they have no conflicts of interest with the contents of this article.
